# Cognitive-Enhancing Effect of* Aronia melanocarpa* Extract against Memory Impairment Induced by Scopolamine in Mice

**DOI:** 10.1155/2016/6145926

**Published:** 2016-04-30

**Authors:** Hyeon Yong Lee, Jin Bae Weon, Youn Sik Jung, Nam Young Kim, Myong Ki Kim, Choong Je Ma

**Affiliations:** ^1^Department of Food Science and Engineering, Seowon University, Cheongju 361-742, Republic of Korea; ^2^Department of Medical Biomaterials Engineering, College of Biomedical Science, Kangwon National University, Chuncheon 200-701, Republic of Korea; ^3^Institute of Bioscience and Biotechnology, Kangwon National University, Chuncheon 200-701, Republic of Korea

## Abstract

*Aronia melanocarpa* (*A. melanocarpa*)* berries* are a fruit with a marked antioxidant effect. The objective of this study was to confirm the effect of* A. melanocarpa berries* extract against scopolamine-induced memory impairment in mice using the Morris water maze and passive avoidance test. Moreover, we determined a possible mechanism of the cognitive-enhancing effect involving AChE activity and BDNF and p-CREB expression in the hippocampus of mice.* A. melanocarpa berries* extract attenuated the learning and memory impairment induced by scopolamine in the Morris water maze (79.3 ± 0.8 s of 200 mg/kg and 64.4 ± 10.7 s of 400 mg/kg on day 4) and passive avoidance tests (46.0 ± 41.1 s of 200 mg/kg and 25.6 ± 18.7 s of 400 mg/kg).* A. melanocarpa berries* extract reduced the acetylcholinesterase level in the hippocampus of scopolamine-injected mice and increased BDNF and p-CREB expression in the hippocampus. The major compound, cyanidin-3-O-galactoside, also reversed memory impairment. These results showed that* A. melanocarpa berries* extract improved memory impairment by inhibiting AChE and increasing BDNF and p-CREB expression, and cyanidin-3-O-galactoside may be responsible for the effect of* A. melanocarpa berries* extract.

## 1. Introduction

Alzheimer's disease (AD) is a progressive neurodegenerative disease that results in memory loss and cognitive dysfunction and is the most common cause of dementia [1]. The symptoms of AD are generally cognitive and memory loss. AD occurs in people over 65 years of age and is associated with various risk factors, including increasing age, family history, and genetic factors.

A characteristic pathogenesis, extracellular deposition of amyloid plaque deposits (A*β*), neurofibrillary tangles (NFT), cholinergic deficiency, and neuronal cell death, is seen in the brains of AD patients [2–4].

Acetylcholine (ACh) is a primary neurotransmitter in interneurons of the central nervous system (CNS) and ACh dysfunction is responsible for memory and cognitive function impairment in the CNS by cholinergic system deficiency [5]. Acetylcholinesterase (AChE) hydrolyses ACh to choline and acetic acid. Excessive AChE activity terminated the Ach before it reaches the ACh receptor [6]. Therefore high level of AChE can be not good for memory function.

Most current AD treatments focus on the inhibition of AChE activity. Indeed, AChE inhibitors, including donepezil, galantamine, tacrine, and rivastigmine, can attenuate memory impairment by inhibiting the destruction of ACh [7, 8].

Brain-derived neurotrophic factor (BDNF) is a member of the neurotrophin family and regulates cell growth in the CNS. It is essential for memory processes and long-term memory formation at the hippocampal and cortical synapse [9].

cAMP-response element binding protein (CREB), a cellular transcription factor, upregulates specific target genes, including BDNF. BDNF and CREB contribute to long-term potentiation (LTP) formation at the hippocampal and cortical synapse. BDNF gene expression is downregulated in the brains of patients with AD [10, 11].

*Aronia melanocarpa berries* (Rosaceae family) are a fruit known as black chokeberry and have a high content of polyphenols, including anthocyanins (cyanidin glycosides), flavanols, flavonoids (quercetin glycosides), chlorogenic acids, triterpenes, and fibers and caffeic acid derivatives [12].* A*.* melanocarpa berries* exhibit high antioxidant activity and had hepatoprotective, gastroprotective, and anti-inflammatory effects [13]. Previous studies have demonstrated that* A. melanocarpa berries* reduce systolic and diastolic blood pressure [14] and protect against female skeleton damage due to chronic exposure to Cd [15].* A. melanocarpa berries* also prevent obesity in C57BL/6J mice [16].* A. melanocarpa* fruit juice improves memory in male Wistar rats on passive avoidance test [17, 18]. However, we performed Morris water maze test and passive avoidance test on scopolamine-induced memory impairment mouse model. In addition, we revealed mechanism of cognitive-enhancing effect.

Scopolamine is a well-known muscarinic cholinergic receptor antagonist (anticholinergic drugs) that causes memory impairments in animals and humans by decreasing central cholinergic activity. Thus, scopolamine-induced learning and memory deficits have been used as a model to screen drugs for potential therapeutic usefulness [19]. There is difference between pathogeneses of Wistar rat model and scopolamine-induced memory impairment model. Various memory impairment models have made significant contributions to better understand the complex pathology of AD.

In the present study, we revealed the effect of* A. melanocarpa berries* extract and cyanidin-3-O-galactoside on scopolamine-induced memory deficits in the Morris water maze and passive avoidance tests. Furthermore, AChE activity and BDNF expression and CREB phosphorylation in the hippocampus of mice were evaluated to determine and understand the mechanism.

## 2. Material and Methods

### 2.1. Plant Materials and Extraction

*A. melanocarpa* sample was manufactured and obtained from Future Food Research Center (Cheng Ju, Korea).* A. melanocarpa* fruit from the 14-year-old plant was collected from Danyang, Chungcheongbuk-do, Korea.* A. melanocarpa berries* were dried for three days in freeze dryer (PVTFA 10AT, ILSHIN BioBase, Dongducheon, Korea) and dried* A. melanocarpa berries* were pulverized using a blender. Powered* A. melanocarpa berries* were extracted in 70% ethanol (100 g/1 L) using maceration at room temperature. This* A. melanocarpa berries* extract was filtered through vacuum filter and concentrated by evaporation (EYELA N-1000, Tokyo Rikakikai Co., Tokyo, Japan).* A*.* melanocarpa berries* concentrate was dried using freeze dryer for three days and powdered.

### 2.2. HPLC Analysis

The HPLC analysis was performed on Dionex system (Dionex, Germany) composed of a pump (LPG 3X00), an autosampler (ACC-3000), a column oven (TCC-3000SD), and diode array UV/VIS detector [DAD-3000(RS)]. Separation was conducted on Phenomenex Luna C18 column (4.6 mmI.D. × 150 mm, 5 *μ*m) at column temperature 30°C.

**Figure 1:**
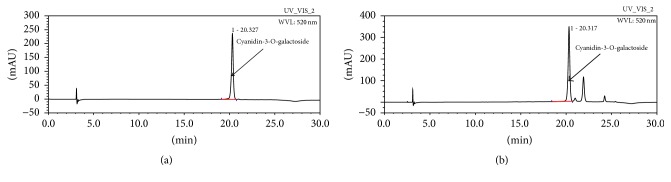
HPLC chromatogram of cyanidin-3-O-galactoside (a) and* A. melanocarpa* berries sample (b).

The mobile phase was composed of (A) HCOOH-H_2_O (1 : 9 v/v) and (B) HCOOH-MeOH-H_2_O (10 : 50 : 40, v/v) and gradient system was as follows: 0% B at 0–2 min, 0–70% B at 2–20 min, 70–100% B at 20–22 min, 100–0% B at 22–24 min, and 0% B at 24–30 min. Flow rate was 0.8 mL/min and sample injection volume was 10 *μ*L. 520 nm of UV spectra was selected for determination of cyanidin-3-O-galactoside in* A. melanocarpa berries* extract. HPLC chromatogram of* A. melanocarpa berries* is shown in Figure 1.

### 2.3. Animals

Male ICR mice aged 5 weeks (25–30 g) were purchased from Daehan Biolink. Co., Ltd. (Chungbuk, Korea). Mice (7 per cage) were maintained in a room at 20 ± 3°C under a 12/12 h light-dark cycle and provided food and tap water* ad libitum* (commercial pellets). All procedures involving animal experiments in this study were performed in accordance with the guidelines of Kangwon National University IACUC (KIACUC: KW-150706-1).

### 2.4. Medicines Administration

Mice were divided into six groups—the control group, scopolamine group, positive control group (donepezil (1 m/kg) treatment), two* A. melanocarpa berries* extract- (200 and 400 mg/kg) treated groups, and a cyanidin-3-O-galactoside- (50 mg/kg) treated group.* A. melanocarpa berries* extract, cyanidin-3-O-galactoside, and donepezil were dissolved in 0.5% CMC solution and scopolamine was dissolved in normal saline.

*A. melanocarpa berries* extract, cyanidin-3-O-galactoside, and donepezil were administered orally to mice 120 min before the water maze test and passive avoidance test. The control and scopolamine groups received 0.5% CMC solution. Scopolamine was administered subcutaneously in all groups with the exception of the control group (normal saline treatment) 30 min before water maze test and passive avoidance test.

Medicine was administered daily for 4 days prior to the Morris water maze test and once for training in the passive avoidance test.

### 2.5. Morris Water Maze Test

The water maze test was performed using the previously described Morris method [20]. The water maze apparatus is a large circular pool (90 cm diameter and 40 cm height) and water (20 ± 1°C) was filled with white milk to a depth of 30 cm. The water maze area was divided into four equal quadrants, and a white escape platform (10 cm diameter and 26 cm height) was located in the center of one quadrant (1 cm above the water surface) and unchanged during the water maze test. Swim starting positions did not use the edge of the same quadrants during the 4-day test trial. All swimming parameters of mice, that is, swim time, distance, and speed, were monitored and analyzed using a smart (ver 2.5.21) video-tracking system linked to the video camera. The test trial was stopped after the mouse reached the platform, and the escape latency was recorded. Mice were trained for 60 s in the absence of the platform on the first day. The mice underwent four trial sessions per day during four consecutive days. The trial sessions were separated by 1 day. Trial sessions were stopped if the mouse did not find the platform within 120 s, and an escape latency of 120 s was recorded. A probe trial was conducted for 60 s on the water maze 24 h after the final test trial. The time spent in the target quadrant was recorded to assess the spatial memory retention of the mouse.

### 2.6. Passive Avoidance Test

The passive avoidance test was carried out using two equally sized light and dark compartments (17 × 12 × 10 cm) with an electrifiable grid floor [21]. The two compartments were separated by a guillotine door. The mouse was initially placed in and explored the light compartment for 40 s in an acquisition trial. When the door was opened, the mouse moved into the dark compartment and the door closed automatically. The training trial was performed 24 h after the acquisition trial. Mice explored the light compartment for 40 s and the guillotine door was opened. As soon as the mice entered the dark compartment, the door closed automatically and an electric foot-shock (0.1 mA/10 g body weight for 2 s) was delivered through the grid floor. At 24 h after the training trial, a test trial was performed using the same training program. The latency prior to mice entering to the dark compartment after opening of the door within 180 s was measured. If mice did not move into the dark compartment within 180 s, a latency time of 180 s was recorded.

### 2.7. Acetylcholinesterase Inhibition Assay

The assay of AChE activity was performed using Ellman's spectrophotometric method [22]. The hippocampi were rapidly isolated from the brains of mice within 30 min after behavior testing and homogenized in sodium phosphate buffer. Thirty-three microliters of supernatant, 470 *μ*L of phosphate buffer (pH 8), and 167 *μ*L of 5,5′-dithiobis(2-nitrobenzoic acid) (3 mM) were mixed and incubated at 37°C for 5 min. Then, 280 *μ*L of acetylcholine iodide (1 mM) was added to the reaction mixture, which was incubated at 37°C for 5 min. AChE activity was measured at a wavelength of 412 nm using a spectrophotometer.

### 2.8. Tissue Preparation and Western Blot Analysis

Hippocampi were prepared from mouse brain within 30 min after behavior testing and promptly homogenized in 200 *μ*L of ice-cold RIPA buffer containing a protein inhibitor cocktail. The homogenates were centrifuged (13,000 ×g for 20 min), and the supernatants were stored at −80°C. The supernatants (40 *μ*g total protein) were subjected to 15% SDS-PAGE gel and transferred to PVDF membranes. The membranes were blocked in 5% skim milk for 1 h and incubated with primary antibodies, mouse anti-*β*-actin (1 : 2000), mouse anti-CREB (1 : 1000), goat anti-pCREB (1 : 500), and rabbit anti-BDNF (1 : 1000), overnight at 4°C. After incubation, membranes were washed three times for 15 min each and incubated with secondary antibodies (goat-anti-rabbit IgG HRP 1 : 2000 for BDNF and horse-anti-goat IgG HRP for pCREB and goat-anti-mouse IgG HRP 1 : 2000 for *β*-actin and CREB) for 1 h at room temperature; the membrane was then washed three times. Detection was performed using an enhanced chemiluminescence (ECL) solution and exposure to X-ray film.

### 2.9. Statistical Analysis

Data are expressed as means ± SEM. The escape latency time, mean distance, swimming speed, and time spent in the target quadrant of the probe test of the Morris water maze test and the latency time of the passive avoidance test, AChE activity values, and western blotting results were subjected to one-way analysis of variance (ANOVA) followed by Tukey's post hoc test. A value of *p* < 0.05 was considered to indicate statistical significance.

## 3. Results

### 3.1. Effect of* A. melanocarpa Berries* Extract on the Morris Water Maze Test

**Figure 2:**
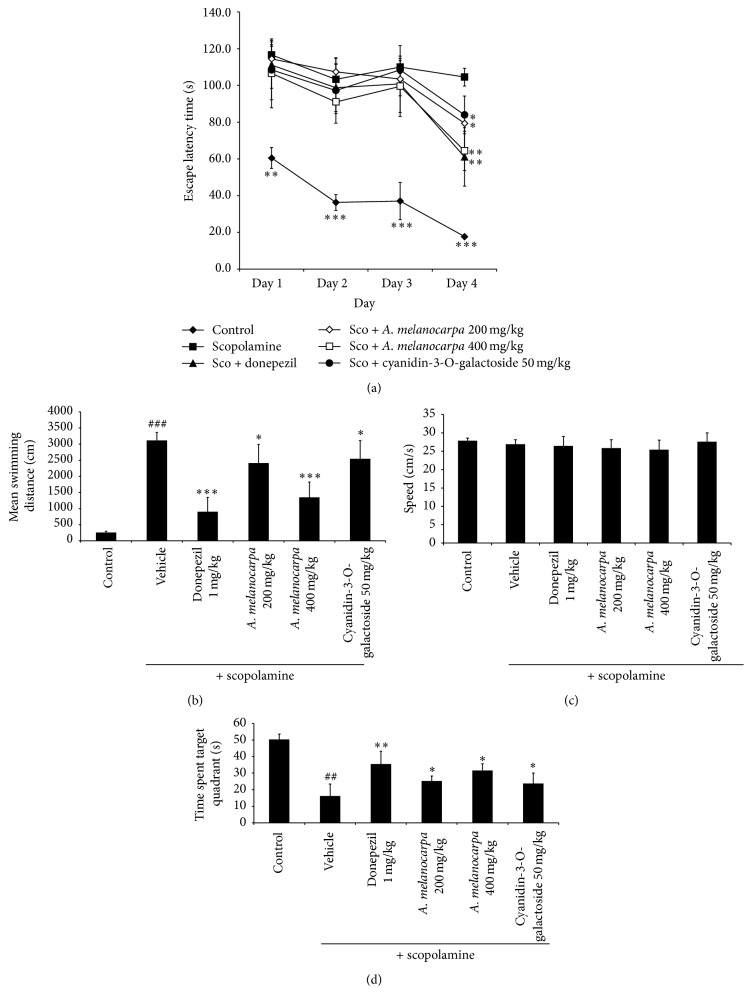
(a) Effect of* A. melanocarpa* berries extract on the escape latency of scopolamine-treated mice in the Morris water maze test.* A. melanocarpa* berries extract (100 and 200 mg/kg body weight, PO), cyanidin-3-O-galactoside (50 mg/kg body weight, PO), and donepezil (1 mg/kg body weight, PO) were administered 90 min before induction of memory impairment by scopolamine. The escape latency of each group during the training-session trials is presented. (b) Mean distance and (c) swimming speed to find the platform over 4 days. (d) Effect of* A. melanocarpa* extract in the probe trial. The time spent in the target quadrant during the probe trial is presented. Data are mean escape latencies ± SD (*n* = 7). (^##^ *p* < 0.01 and ^###^ *p* < 0.001 versus the control group; ^*∗*^ *p* < 0.05, ^*∗∗*^ *p* < 0.01, and ^*∗∗∗*^ *p* < 0.001 versus scopolamine-treated mice.) Sco: scopolamine.

The effect of* A. melanocarpa berries* extract (200 and 400 mg/kg) on scopolamine-induced spatial memory impairment was evaluated by means of the Morris water maze test (Figure 2(a)). The escape latency (s) of the control group decreased significantly over the 4 trial days. In contrast, the escape latency in the scopolamine-treated group was unchanged after day 1. This indicated that scopolamine induced memory impairment. The* A. melanocarpa berries* extract- (200 and 400 mg/kg) treated groups showed significantly decreased escape latencies (s) on day 3 and day 4 (*p* < 0.05). There was significant difference between the 200 and 400 mg/kg* A. melanocarpa berries* extract groups on day 4. Cyanidin-3-O-galactoside (50 mg/kg) significantly decreased escape latency to a level similar to that of 200 mg/kg of* A. melanocarpa berries* extract on day 4. The donepezil-treated group also exhibited significantly decreased escape latency (s) after 4 days. The mean swimming distance to reach the platform on day 4 was exhibited (Figure 2(b)). The* A. melanocarpa berries* extract- and cyanidin-3-O-galactoside-treated group exhibited a significantly decreased swimming distance compared to the scopolamine-treated group (*p* < 0.05). The mean swim speed of mice in all groups during 4 days was not significantly different (Figure 2(c)), suggesting that the treatments did not affect the locomotor activity of mice.

In the probe test on the final day, the time spent in the target quadrant was higher in the scopolamine-treated group compared to the control group. The* A. melanocarpa berries* extract- and cyanidin-3-O-galactoside-treated group showed a significantly increased time spent in the target quadrant compared with the scopolamine-treated group (Figure 2(d)).

### 3.2. Effect of* A. melanocarpa Berries* on the Passive Avoidance Test

**Figure 3:**
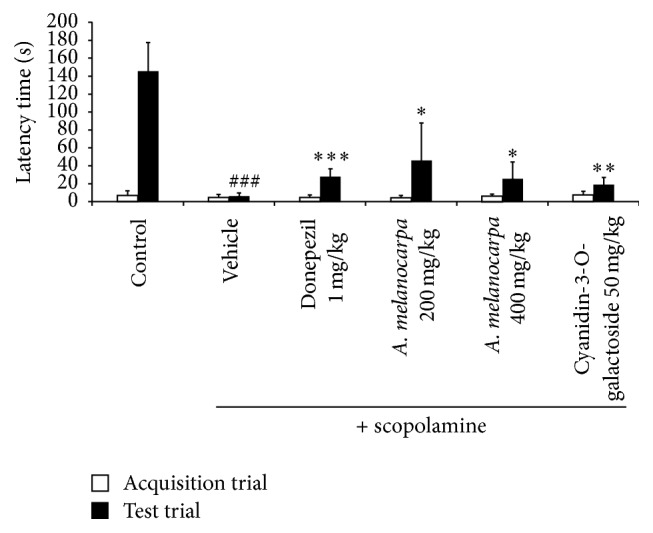
Effect of* A. melanocarpa* berries extract on scopolamine-induced memory impairment in the passive avoidance test. The latency prior to entry to the dark compartment was recorded. Data are mean latency times (s) ± SD (*n* = 7). ^###^ *p* < 0.001 versus the control group; ^*∗*^ *p* < 0.05, ^*∗∗*^ *p* < 0.01, and ^*∗∗∗*^ *p* < 0.001 compared with the scopolamine group.

We investigated the effect of* A. melanocarpa berries* extract on scopolamine-induced memory deficit using the passive avoidance test (Figure 3). There was no significant difference in latency time among the groups in the acquisition trial. The latency time of the scopolamine-treated group was significantly decreased compared with that of the control group. The* A. melanocarpa berries* extract- and cyanidin-3-O-galactoside-treated mice exhibited a significantly ameliorated latency time. Treatment with donepezil, as the positive control, also resulted in a significant increase in latency time.

### 3.3. AChE Inhibitory Effect of* A. melanocarpa Berries* Extract

**Figure 4:**
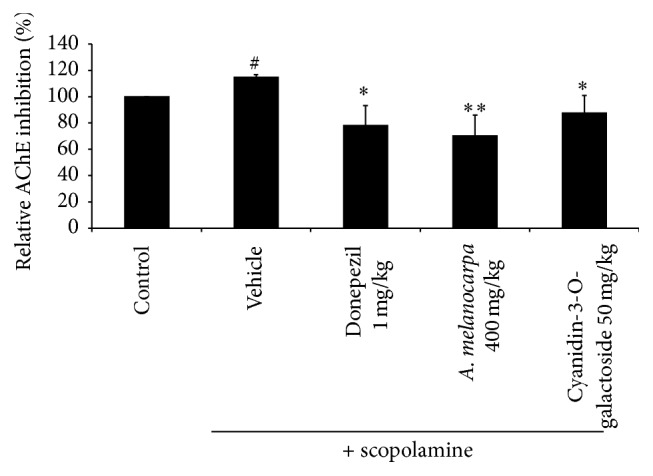
Effect of* A. melanocarpa* berries extract on acetylcholinesterase (AChE) activity in the hippocampi of the mice. Data are means ± SD. ^#^ *p* < 0.05 versus the control group; ^*∗*^ *p* < 0.05 and ^*∗∗*^ *p* < 0.01 compared with thescopolamine-treated group (*n* = 3).

The effect of* A. melanocarpa berries* extract on AChE activity in the hippocampus of scopolamine-induced memory-impaired mice was evaluated. The scopolamine-treated group exhibited a significantly increased AChE level in the hippocampus compared to the control group.* A. melanocarpa berries* extract (400 mg/kg) and cyanidin-3-O-galactoside (50 mg/kg) significantly inhibited AChE activity in the hippocampus by 70.23% and 87.43%, respectively (Figure 4). Treatment with donepezil (positive control) also decreased AChE activity.

### 3.4. Effect of* A. melanocarpa Berries* Extract on BDNF Expression and CREB Phosphorylation

**Figure 5:**
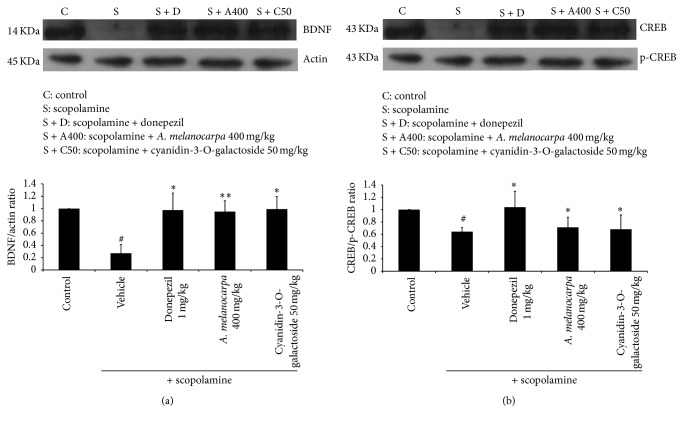
The effect of* A. melanocarpa* berries extract on BDNF and p-CREB expression in the hippocampi of the mice by western blot analysis. Data are means ± SD. ^#^ *p* < 0.05 versus the control group; ^*∗*^ *p* < 0.05 and ^*∗∗*^ *p* < 0.01 compared with the scopolamine-treated group (*n* = 3).

We evaluated the effect of* A. melanocarpa berries* extract on BDNF expression and p-CREB phosphorylation in the hippocampus by western blot analysis (Figure 5).

The expression of BDNF and phosphorylation of CREB (p-CREB) were decreased after exposure to scopolamine compared to the control group.* A. melanocarpa berries* extract-treated mice exhibited significantly increased BDNF and pCREB levels in the hippocampus compared with scopolamine-treated mice.

## 4. Discussion

We evaluated the effect of* A. melanocarpa berries* extract on scopolamine-induced learning and memory deficits using the Morris water maze and passive avoidance tests. The Morris water maze test is designed to assess spatial memory and learning function [20], and the passive avoidance test, a fear-motivated avoidance test, was used to evaluate memory retention [21].

Scopolamine, a nonselective and competitive muscarinic cholinergic receptor antagonist, is widely used in mouse-behavior tests of cognition and memory. Scopolamine induces learning and memory impairment by blocking cholinergic signaling [19].

Escape latency of repeated trial tests for 4 days and the time spent in the target quadrant in the probe test were investigated in the Morris water maze test. These results indicated that* A. melanocarpa berries* extract attenuated scopolamine-induced spatial memory impairment and improved long-term memory in the Morris water maze test.

The passive avoidance test is performed as the animal learns to avoid an aversive stimulus (electrical foot-shock). Administration of* A. melanocarpa berries* extract ameliorated the scopolamine-induced memory deficit in the passive avoidance test, suggesting that it may improve long-term memory.

AChE hydrolyses and inactivates ACh. Increased AChE activity leads to a lack of ACh and thus memory deficits, as observed in the brains of AD patients [23, 24].

Scopolamine increases AChE activity and we investigated the AChE inhibitory effect of* A. melanocarpa berries* extract.* A. melanocarpa berries* extract inhibited AChE activity in the hippocampus of mice.

These results indicated that* A. melanocarpa berries* extract reverses cognitive impairment by increasing cholinergic activity through the inhibition of AChE activity.

BDNF expression and p-CREB phosphorylation play an essential role in memory processes and contribute to LTP formation at the hippocampal and cortical synapse. Phosphorylation of CREB leads to upregulation of BDNF (phosphorylated at Ser-133) [25–27].

We investigated the effect of* A. melanocarpa berries* extract on CREB phosphorylation and BDNF expression in scopolamine-treated mice by western blot analysis. We confirmed that* A. melanocarpa berries* extract increased BDNF expression and CREB phosphorylation in the hippocampi of the mice. These results indicated that the cognitive-enhancing effect of* A. melanocarpa berries* extract correlates with the activation of BDNF and CREB.

In addition, we revealed the effect of cyanidin-3-O-galactoside on AChE activity and BDNF and pCREB expression. The results suggested that cyanidin-3-O-galactoside contributed to the cognitive-enhancing effect of* A. melanocarpa berries* extract.

In conclusion, we determined that* A. melanocarpa berries* extract ameliorates scopolamine-induced memory deficits in mice in the Morris water maze and passive avoidance tests.* A. melanocarpa berries* extract also inhibited AChE activity and activated BDNF and p-CREB signaling. The present study suggests* A. melanocarpa berries* extract to have potential therapeutic agent for the prevention and treatment of neurodegenerative diseases such as AD.

## Abbreviations

*A. melanocarpa*: * Aronia melanocarpa*
AD: Alzheimer's disease
A*β*: Amyloid plaque deposits
NFT: Neurofibrillary tangles
ACh: Acetylcholine
CNS: Central nervous system
AChE: Acetylcholinesterase
BDNF: Brain-derived neurotrophic factor
CREB: cAMP-response element binding protein
p-CREB: Phosphorylation of CREB
LTP: Long-term potentiation
ECL: Enhanced chemiluminescence.
